# Bespoke automated linkage to enable analysis of covid deaths by ethnicity.

**DOI:** 10.23889/ijpds.v7i3.2050

**Published:** 2022-08-25

**Authors:** Shelley Gammon, Rachel Shipsey, Charlie Tomlin, Josie Plachta

**Affiliations:** 1 Office for National Statistics

In early 2020 there was intense media speculation that ethnicity and Covid-19 deaths were correlated. However, the existing method of adding ethnicity to death records resulted in low linkage rates for very recent deaths. We designed and implemented a bespoke linkage in three days enabling accurate reporting to the nation.

We linked the 2011 England and Wales Census to death records using a range of personal identifiers. Due to time pressure, we focused on executing a single linkage method well. Deterministic linkage was chosen, using a variety of matchkeys which were tested via clerical review. To overcome the issue of addresses changing since 2011, we also linked 2020 death record residuals to the 2019 Patient Register (PR) and then made use of the 2011 PR address where it existed. This additionally provided an indication of whether unmatched death records might be attributable to migration into England and Wales post-2011.

The prior linking method used NHS Number only. Although the overall linkage rate was approximately 90%, the rate for recent deaths (2nd March 2020 to 10th April 2020 in the first iteration of the linkage) was closer to 30% due to an administrative lag in adding NHS Numbers to death records. Our novel bespoke linkage method linked over 39,000 extra death records. Whilst this had minimal impact on the overall linkage rate, it improved the linkage rate for recent deaths to approximately 90%. This was without an impact on accuracy: clerical review demonstrated that the false positive rate was approximately 0.2%. A report was published using this data showing that the risk of death involving Covid-19 among some ethnic groups was significantly higher than others.

Determining whether Covid-19 disproportionally affected certain ethnicities was of crucial importance in the early phase of the pandemic to enable appropriate government strategies to be developed. We delivered a bespoke linkage under an exceptional time-limit without compromising on accuracy, enabling this impactful analysis with nation-wide interest and impact.

